# Functional Organization of the Action Observation Network in Autism: A Graph Theory Approach

**DOI:** 10.1371/journal.pone.0137020

**Published:** 2015-08-28

**Authors:** Kaat Alaerts, Franca Geerlings, Lynn Herremans, Stephan P. Swinnen, Judith Verhoeven, Stefan Sunaert, Nicole Wenderoth

**Affiliations:** 1 Department of Kinesiology, Movement Control & Neuroplasticity Research Group, KU Leuven, Leuven, Belgium; 2 Department of Rehabilitation Sciences, Neuromotor Rehabilitation Research Group, KU Leuven, Leuven, Belgium; 3 Department of Imaging & Pathology, Translational MRI, KU Leuven, Leuven, Belgium; 4 Department Health Sciences and Technology, Neural Control of Movement Lab, ETH Zurich, Zurich, Switzerland; Institute of Psychology, Chinese Academy of Sciences, CHINA

## Abstract

**Background:**

The ability to recognize, understand and interpret other’s actions and emotions has been linked to the mirror system or action-observation-network (AON). Although variations in these abilities are prevalent in the neuro-typical population, persons diagnosed with autism spectrum disorders (ASD) have deficits in the social domain and exhibit alterations in this neural network.

**Method:**

Here, we examined functional network properties of the AON using graph theory measures and region-to-region functional connectivity analyses of resting-state fMRI-data from adolescents and young adults with ASD and typical controls (TC).

**Results:**

Overall, our graph theory analyses provided convergent evidence that the network integrity of the AON is altered in ASD, and that reductions in network efficiency relate to reductions in overall network density (i.e., decreased overall connection strength). Compared to TC, individuals with ASD showed significant reductions in network efficiency and increased shortest path lengths and centrality. Importantly, when adjusting for overall differences in network density between ASD and TC groups, participants with ASD continued to display reductions in network integrity, suggesting that also network-level organizational properties of the AON are altered in ASD.

**Conclusion:**

While differences in empirical connectivity contributed to reductions in network integrity, graph theoretical analyses provided indications that also changes in the high-level network organization reduced integrity of the AON.

## Introduction

Autism spectrum disorders (ASD) encompass a group of complex neurodevelopmental conditions affecting communication and social interaction skills. Diagnosis is currently based on behavioral evaluations since neural biomarkers have proven elusive. An influential but controversial theory posits that social deficits in ASD stem from underactivation of the brain’s action observation network (AON) or mirror system [1,2]. Mirror regions have the property to activate both during the execution as well as the observation of movements, and both in the monkey and human brain, the core mirror system constitutes a fronto-parietal network (including inferior frontal gyrus (IFG), premotor cortex and the inferior parietal lobule (IPL)), which receives its main visual input from the superior temporal sulcus (STS) [3]. Based on its unique observation-to-execution matching properties, the mirror system has been linked to several socio-cognitive functions including the understanding of others actions, and a person’s ability to imitate or identify with the actions and emotions of others [4].

To date, a number of neurophysiological techniques, including transcranial magnetic stimulation (TMS), electro-encephalography (EEG), and functional magnetic resonance imaging (fMRI), have been adopted to study the hypothesis of 'broken mirrors' in the autistic brain. While TMS studies provided consistent evidence of altered ‘mirroring’ in the autistic brain [5,6], EEG research revealed evidence of both typical [7,8] and atypical [9–12] mirror system responses in ASD. Also from several task-based fMRI studies, a mixed pattern of results has emerged. Overall, it appears that only in the studies that used emotional stimuli, group differences brain activations were apparent, whereas in the studies that used non-emotional studies, equivalent or even increased mirror responses have been reported (for review see [13]).

More recently, ASD has been suggested to constitute a ‘developmental disconnection’ syndrome, with altered neuronal development leading to reduced connectivity at the systems level [14–17]. In relation to the broken mirror account, several task-based fMRI studies showed reduced functional connectivity in ASD of several brain regions, part of the AON, that are recruited during facial processing tasks [18–21]. For example, Schipul et al. (2012) measured fMRI BOLD responses during a facial social judgment task, and found that inter-regional synchronization of brain activity (i.e., reflecting interregional communication/ connectivity during the task) was decreased in the ASD group compared to the control group [20]. Similarly, Rudie et al. (2012) showed that individuals with ASD displayed significant reductions in connectivity between right IFG and inferior and superior parietal lobules while passively viewing emotional face expressions [21]. Also several other studies reported altered activity patterns and/or differences in functional connectivity of the AON in ASD [22–25]. Particularly, Kana et al. (2012) explored the recruitment of AON areas during an intentional causal attribution task and found that participants with ASD showed lower activation in temporo-parietal junction (TPJ), right IFG and left premotor cortex, as well as reduced functional connectivity between TPJ and motor areas [23]. Shih et al. (2010) explored region-to-region connectivity between IFG, IPL and STS during a semantic decision or letter detection task and although intrinsic synchronicity between regions was less robust in ASD participants compared to typical controls (TC), group differences did not reach significance [24]. Recently, resting-state fMRI emerged as a novel approach to examine *intrinsic* functional network organization, by studying correlations of *spontaneous* fluctuations in the fMRI BOLD-response that are unrelated to a particular task [26,27]. A previous resting-state fMRI study from our lab showed remarkable ASD-related reductions in intrinsic functional connectivity (iFC) between STS and bilateral parietal regions, part of the AON [22]. More recently, Fishman et al. (2014) also explored resting-state iFC of the AON but found no significant overall group differences. Only within an ASD subset sample with most severe symptoms, significant between-group differences were revealed, indicative of greater connectivity between the right anterior inferior parietal sulcus and left superior frontal gyrus and posterior cingulate cortex in the ASD-group compared to the TC-group [25].

While most resting-state fMRI studies adopt region-of-interest (ROI) based approaches to assess differences in the strength of correlations between distinct regions within a particular network, some initial studies have used graph theory analyses [28] to examine alterations in higher-level whole-network intrinsic functional organization in autism. Rudie et al. (2012) used resting-state fMRI to explore graph theoretical metrics within a whole-brain 264-region functional parcellation scheme and showed network-level reductions in modularity and clustering (local efficiency), as well as shorter characteristic path lengths (higher global efficiency) in ASD [29]. Redcay et al. (2013) assessed graph theoretical metrics of 34 regions encompassing four intrinsic ‘resting-state’ networks (the cingulo-opercular, cerebellar, fronto-parietal and default network), but in this study, only minimal group differences in graph measures were revealed [30], indicating that overall organizational properties of these networks are intact in ASD.

In relation to the broken mirror theory of autism, the present study specifically explored graph theoretical properties of the AON using resting-state fMRI data of adolescents and young adults with ASD. Network analyses were performed using 14 bilateral nodes of the extended AON in the frontal, parietal, and temporal lobes, identified from previous task-based fMRI studies on action observation [22,31]: inferior frontal gyrus (IFG), premotor cortex (PMC), inferior parietal lobule (IPL), intraparietal sulcus (IPS), primary somatosensory cortex (SI), superior parietal lobule (SPL), fusiform gyrus (FG), and superior temporal sulcus (STS). Global efficiency, local efficiency, betweenness-centrality, average path length, degree, and cost were assessed as high-level network properties. Also basic region-to-region network connections were assessed using the same 14 ROIs constituting the AON.

In addition to the exploration of ASD-TC group differences, we specifically aimed to explore whether measures of functional network organization of the AON are correlated to behavioral performance on a bodily emotion recognition task probing whether an emotional state can be inferred from whole body kinematics as depicted by point light displays (PLD) [22,32,33]. Paradigms involving PLD perception have been shown sensitive for revealing deficits in toddlers, children and adults with ASD (for review see [34]) and prior work showed that the processing of PLDs is mediated by regions of the AON [35–37]. Salient processing of biological motion is a critical aspect of typical social behavior [38–42,42] and several prior task-based fMRI studies examining the neural correlates of PLD-perception in ASD consistently showed altered activations in regions of the AON [22,40,43–46]. With the assessment of brain-behavior correlations, we aim to provide novel insights into the relationship between inter-individual variations in the intrinsic functional network organization of the AON and behavioral variations in ‘reading’ PLD motion with emotional content.

## Methods

### Ethics Statement

The study was approved by the local Ethical Board at the KU Leuven and written informed consent was obtained from all participants or their parents/guardians according to the Declaration of Helsinki.

### Participants

Resting-state fMRI data were collected from 27 participants with ASD (2 females) and 31 typical controls (TC) (3 females). Table 1 provides descriptive phenotypic data on the ASD- and TC-groups. The sample included adolescent (ages 12–17 years) (12 ASD, 16 TC) and adult participants (ages 18.5–25 years) (15 ASD, 15 TC). Resting-state fMRI data were collected at the KU Leuven and were contributed to the online data-sharing repository ABIDE (Autism Brain Imaging Data Exchange (ABIDE)); ‘Sample Leuven 1’ (adults) and ‘Sample Leuven 2’ (adolescents)) [47]. The adult participants were part of the sample previously described in [22]. S1 Table provides descriptive phenotypic data separately for the adolescent and adult participants.

**Table 1:** Group characteristics for the ASD and TC participants (adolescents and adults combined).

	ASD (n = 27)		TC (n = 31)			
Sex	25 males		28 males			
	mean	SD	mean	SD	t-value	p-value
SRS Raw Total Scores	93.3	28.3				
Age (years)	18.2	5.0	18.6	5.1	-0.3	0.75
Verbal IQ	100.7	16.8	116.6	11.0	-4.3	< .001
Performance IQ	103.3	16.2	107.9	13.7	-1.2	0.20
Head Motion (mean FD)	0.13	0.08	0.14	0.05	-0.07	0.95

ASD. autism spectrum disorder; TC. typical controls; SRS. Social Responsiveness Scale; IQ. intelligence quotient; mean FD. mean framewise displacement; SD. standard deviation.

ASD- and TC-participants were matched for age, gender, and performance IQ (Table 1). Verbal IQ was significantly lower in the ASD-group; for adolescents, assessed using the abbreviated version of the Dutch Wechsler Intelligence Scale for Children, Third Edition (WISC-III-NL) (Kort et al., 2005); for adults, assessed using the Ward 7-subtest of the Wechsler Adult Intelligence Scale-III [48–50].

All ASD-participants were recruited from the Autism Expertise Centre at the Leuven University Hospital. A multidisciplinary team (child psychiatrist and/or expert neuro-paediatrician, psychologist, speech/language pathologist and/or physiotherapist) formulated a DSM-IV-TR diagnosis of autistic disorder [51]. Diagnosis was obtained by combining information from unstructured direct observation, semi-structured parent interview (Developmental, dimensional and diagnostic interview (3di)), [52], as well as review of prior history and parent screening questionnaires. Parents completed the Dutch version of the Social Responsiveness Scale (Roeyers et al., 2007) [53], a 65-item questionnaire developed to assess a wide range of interpersonal behavior, communication and repetitive/stereotypic behavior characteristic of ASD [54,54,55]. Only participants with an ASD-diagnosis, and a parental total SRS-score (raw) above 60, were included in the study.

### Behavioral assessments

For each participant, behavioral performance on a bodily emotion recognition task based on point light displays was assessed to explore brain-behavior relationships.

All participants completed two runs, each consisting of three blocks of the emotion recognition task, interleaved with blocks of a control task. Each task block consisted of 5 trials, making a total of 30 trials (2 runs x 3 blocks x 5 trials) for each task (emotion, control).

**Fig 1:**
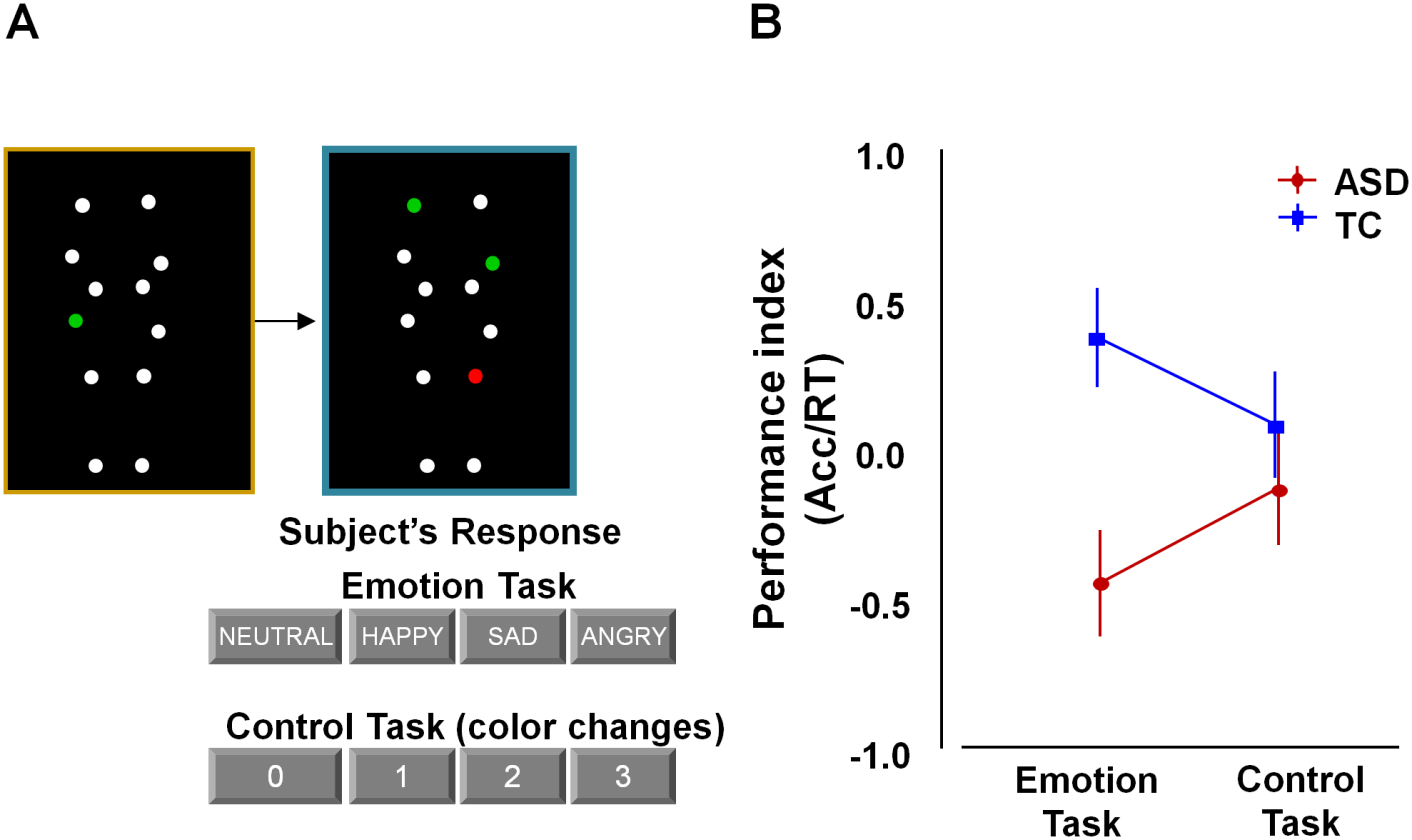
Behavioral performance on the bodily emotion recognition task. Participants determined the emotional state of point light displays (PLD) in which moving white dots reflected the main joints of the human body. (A) The emotional state of the blue-bordered PLD had to be indicated relative to the baseline yellow-bordered PLD (always showing a neutral emotional state). The same PLD-stimuli were presented in a four-choice control task matched for cognitive and motor demands. Here, one of the dots in the yellow-bordered PLD briefly changed color to either red or green. Subsequently, participants had to indicate the number of dots that changed into the same color in the blue-bordered PLD (2 in this example). (B) Performance was higher in the TC-group compared to the ASD-group on the emotion recognition task, but not on the control task.

In both tasks, stimuli consisted of moving point light displays (PLD) as previously described [22,32,33]. In short, twelve reflective markers attached to the joints of the ankles, knees, hips, wrists, elbows and shoulders of human actors were tracked using an eight-camera VICON system (Oxford Metrics, UK). In the resulting 3 sec movies, markers were visible as moving white spheres on a black background (Fig 1A, S1 Movie). The stimuli portrayed human actions (walking; jumping; kicking) that express four bodily emotional states: anger, happiness, sadness or neutral.

In the **emotion recognition task** (S1 Movie), each trial showed a yellow-bordered PLD (3 sec movie), followed by a blue-bordered PLD (3 sec movie), followed by a 2 sec response time period showing a black screen. Participants were asked to indicate whether the presented point-light figure in the blue-bordered movie showed a different emotional state compared to the point light figure in the yellow-bordered movie. The emotional state of the blue-bordered PLD could either be indicated as happier, sadder, angrier, or not different (neutral) from the yellow-bordered PLD (Fig 1A). The yellow-bordered movie always showed a point-light figure in the ‘neutral emotional state’, whereas the emotional state of the blue-bordered point-light figure could either be neutral (7 of 30 trials), happy (7/30), sad (8/30), or angry (8/30).

In the **control task** (S2 Movie), participants were presented with exactly the same set of movies as those presented during the emotion recognition blocks, albeit in a different order and with a different task instruction. Instead of focusing attention on the emotional content in the PLD movies, participants were instructed to indicate color-changes in the PLDs. In the yellow-bordered PLD, one dot briefly (0.5 sec) changed color to ‘red’ or ‘green’ at a random time-point. Participants then had to indicate the number of dots (0-1-2-3) that changed into the same color in the blue-bordered PLD (Fig 1A).

Participants were instructed to respond as fast and accurately as possible.

For each task, reaction times (RTs) and accuracy rates (% correct answers) were assessed using E-prime 2.0 professional (Psychological Software Tools). A single performance index was calculated by dividing accuracy scores by RTs (accuracy/RTs). RTs recorded from the correct trials were considered as outliers and removed from the analysis when they exceeded Q3±[1.5x(Q3-Q1)] with Q1 and Q3 denoting the first and third quartile over the whole set of correct trials for each subject (Statistica, StatSoft, Tulsa, USA). According to these criteria, only a small percentage of trials across all subjects were excluded from the analysis [emotion recognition task: 0.28%; control task: 0.57%]. On both tasks, the number of excluded trials was comparable for both groups.

#### Statistics

Between-group differences in performance (accuracy, RT, performance index) were explored using a 2 x 2 repeated measures ANOVA analysis with ‘group’ (ASD, TC) as a between-subject variable and ‘task’ (emotion, control) as a within-subject repeated-measures variable. Statistics were performed using Statistica 9.0 (StatSoft Tulsa, USA.).

### MRI Data Acquisition

Neuroimaging was performed using a 3.0 Tesla Philips MR-scanner (Best, The Netherlands) with an 8-channel phased-array head-coil.

**Anatomical imaging** consisted of a high-resolution structural volume acquired using a coronal three-dimensional turbo field echo T1-weighted sequence with the following parameters: 182 contiguous coronal slices covering the whole brain and brainstem, slice thickness = 1.2 mm; repetition time (TR) = 9.7 ms; echo time (TE) = 4.6 ms; matrix size = 256 x 256; field-of-view (FOV) = 250 x 250 mm^2^; in-plane pixel size = 0.98 x 0.98 mm^2^; acquisition time = 6 min 38 s.

**Resting-state fMRI** images were acquired using a T2*-weighted gradient-echo echo planar imaging (GE-EPI) sequence with the following parameters: TR = 1700 ms; TE = 33 ms; matrix size = 64 x 64, FOV = 230 mm; flip angle 90°; slice thickness = 4 mm, no gap; axial slices = 32; 250 functional volumes; acquisition time = 7 min. Participants were instructed to relax (but not sleep), keep their eyes open while staring at a white cross and think of nothing in particular during the resting-state fMRI scanning.

### Image preprocessing

SPM-8 (Wellcome Department of Imaging Neuroscience, London, UK) and the CONN functional connectivity toolbox [56] were used for image preprocessing and statistical analyses implemented in Matlab R2008a (Mathworks).

Resting-state fMRI images were spatially realigned, corrected for differences in slice acquisition time by temporal interpolation to the middle slice (reference = 17), normalized to the standard EPI-template of the Montreal Neurological Institute (MNI-152), resampled into 3-mm isotropic voxels and spatially smoothed with an isotropic 5-mm full-width-at-half-maximum Gaussian kernel.

Resting-state images were band-pass filtered (0.009 < f < 0.08Hz) and six realignment parameters were modeled as regressors of no-interest. White matter and cerebrospinal fluid were also removed as confounds following the implemented CompCor-strategy [57] in the CONN functional connectivity toolbox. No global signal regression was applied.

Mean frame-wise displacement (FD) scores did not exceed 0.5 mm in any of the participants and were not significantly different between groups (Table 1, S1 Fig). Nonetheless, considering that even small amounts of movement can produce spurious intrinsic functional connectivity (iFC) [58–60], we accounted for inter-individual differences in micro-movements by including mean FD-scores as a nuisance covariate at the group-level in all analyses. Furthermore, all analyses were performed on ‘scrubbed’ data [61], i.e., censoring frames displaying FD>0.5 mm or frame-wise changes in brain image intensity exceeding >0.5 Δ%BOLD. In none of the subjects, the proportion of scrubbed volumes exceeded 50%. Also no significant group difference was revealed in the proportion of scrubbed volumes [ASD: 6.5 ± 12%; TC: 4 ± 4.6%; t(56) = 1.0, p = 0.3].

### Graph theoretical measures

Graph theory analyses were performed for regions of the action observation network (AON) (Table 2) using the CONN functional connectivity toolbox.

**Table 2:** Regions of interest of the action observation network.

Number		Macroanatomical location	Cytoarchitectonic location	MNI coordinates		
				*x*	*y*	*z*
1	L	IFG / PrG	BA 44 / BA 45 / vent-lat BA 6	-50	9	30
2	R	IFG	BA 44	52	12	26
3	L	lat dPMC	dors-lat BA 6	-26	-4	56
4	R	lat dPMC / MFG	dors-lat BA 6	34	-2	54
5	L	IPL	PFt / PFop	-60	-24	36
6	R	IPL	PFt	44	-34	44
7	L	SI / IPS / SPL	BA 2 / hIP3 / 7A	-34	-44	52
8	R	SI	BA 1 / 2	60	-20	40
9	R	IPS	hIP3	30	-54	48
10	R	SPL	7A	22	-62	64
11	L	fusiform gyrus		-44	-56	-18
12	R	fusiform gyrus		44	-54	-18
13	L	pSTS / pMTG		-47	-60	4
14	R	pSTS / pMTG		47	-60	4

Numbers correspond to the regions shown in Fig 3. L: left, R: right, IFG: inferior frontal gyrus, PrG: precentral gyrus, dPMC: dorsal premotor cortex, MFG: middle frontal gyrus, IPL: inferior parietal lobule, SI: primary somatosensory cortex, IPS: intraparietal sulcus, SPL: superior parietal lobule, pSTS: posterior superior temporal sulcus, pMTG: posterior middle temporal gyrus, BA: brodmann area.

Twelve regions-of-interest (ROIs) for the core fronto-parietal AON were defined based on a recent meta-analysis of action observation studies [31]. Left and right posterior superior temporal sulcus (pSTS) ROIs were based on [22] representing important input areas to the AON. In total, 14 ROI spheres (radius 10 mm) were included constituting a bilateral temporo-fronto-parietal network (Table 2).

For each subject, the residual BOLD time-course was extracted from each ROI and bivariate correlation coefficients between its time-course and the time-course of all other ROIs was computed. The ROI-to-ROI correlation matrix was then thresholded to construct a binary matrix where existing (valid) connections were assigned a value of 1, while the absence of a functional connection between network nodes was designated by a value of 0. Self-connections of nodes were not included in the analyses.

Two approaches were applied to threshold the graph theoretical network.

The first approach is consistent with the approach used in the study by Redcay et al. (2013). Here, graph networks were constructed using a fixed cost threshold, ensuring that the density or number of connections of the network is equated across all individuals and groups. Cost is a measure of the proportion of connections for each ROI in relation to all connections in the network. The use of a fixed cost threshold allows for roughly equal numbers of connections across participants by varying the correlation (r-value) threshold for each participant to achieve the fixed cost threshold. As there is no rationale for using a particular cost threshold to determine whether a connection exists in a functional network, we compared graph network properties for a wide range of cost thresholds, ranging from *k* = 0.15 to *k* = 0.50 with an interval of 0.01 (total of 36).

We additionally used an alternative thresholding approach, based on fixed correlation value thresholds, as adopted frequently in other graph theoretical studies [62–68]. Here, network density was not a priori equated across groups, but instead, the ROI-to-ROI correlation matrices were thresholded using a fixed correlation value threshold. Considering that the choice of correlation value and hence the number of included connections can have a noticeable impact on graph theoretical measures, data were examined using a wide range of correlation value thresholds. Particularly, for each subject, ROI-to-ROI correlation matrices were thresholded at fixed correlation values starting at *r* = 0.12(5) to *r* = 0.50 with an interval of 0.01 (total of 39). This provided binary matrices with overall increasingly smaller connection densities. Note that *r* = 0.125 corresponds to the lowest r-value ensuring significant correlations at p < 0.05. All employed thresholds were one-sided, such that only positive correlations are considered.

The resulting thresholded adjacency matrices from both approaches (36 fixed cost thresholds; 39 fixed correlation value thresholds) served as principal input to calculate the graph theoretical measures.

Measures of interest were global efficiency; local efficiency; betweenness-centrality: average path length; degree; and cost. **Degree** and **Cost** are defined as the number and proportion, respectively, of connections for each node (ROI) to all other nodes in the network. **Global efficiency** is defined as the average inverse shortest path length from one node to all other nodes in the graph. The shortest path length is defined as the fewest number of connections (or correlations) between two nodes. Thus, a network with high global efficiency would be one in which nodes are highly integrated so the path length between nodes is consistently short. The **average (shortest) path length** was defined as the average number of steps along the shortest paths for all possible pairs of network nodes. **Local efficiency** is defined as the average global efficiency within a local subgraph consisting only of the neighbors of a given (index) node. In other words, local efficiency can be understood as a measure of the fault tolerance of the network, indicating how well each subgraph exchanges information when the index node is eliminated. We also examined **betweenness-centrality**, which measures the fraction of all of the shortest paths in a network that contain a given node, with higher numbers indicating participation in a large number of shortest paths. For the ASD and TC groups, nodes with the largest betweenness-centrality were identified as ‘hubs’ in the network if the values of nodal betweenness were 2 SDs greater than the average betweenness-centrality of the network [69].

#### Statistics

Between-group differences in the graph measures (global efficiency, local efficiency, centrality, path length, cost and degree) were explored using repeated measures ANOVA analyses with ‘group’ (ASD, TC) as a between-subject variable and ‘graph threshold’ as a within-subject repeated-measures variable. A 2 x 36 ANOVA model was constructed for the graph measures with cost thresholds; whereas for the graph measures with correlation value thresholds, a 2 x 39 ANOVA model was constructed. Note that group differences in degree and cost were only explored for the correlation value thresholded graphs since these measures are fixed across participants (and groups) for graphs with fixed cost thresholds. For all group analyses, the following nuisance regressors were included in the model: head mircro-motion (mean FD), performance IQ, age and sample (adolescent sample or adult sample).

In addition to the exploration of between-group differences, we explored whether brain-behavior relationships exist between emotion recognition performance and any of the graph measures. To do so, a general linear model was constructed with the regressor of interest ‘emotion recognition performance index’ and head mircro-motion (mean FD), performance IQ, age and sample as nuisance regressors. The within-subject repeated measures variable ‘graph threshold’ was also included in the model to examine whether brain-behavior relationships varied depending on the applied graph threshold (36 for the cost threshold analysis; 39 for the correlation value threshold analysis). Brain-behavior relationships were explored continuously across all ASD- and TC-participants to explore whether inter-individual variations in functional network organization (irrespective of group) are related to behavioral variations in ‘reading’ body language with an emotional content.

All statistics were performed using Statistica 9.0 (StatSoft Tulsa, USA.).

### ROI-to-ROI intrinsic functional connectivity

In addition to the graph theory analyses, we performed resting-state region-of-interest ‘ROI-to-ROI’ iFC analyses using the CONN functional connectivity toolbox with the same 14 ROIs as used for the graph theory analysis (Table 2). For each subject, ROI-to-ROI correlation coefficients were computed and converted to normally distributed z-scores using Fisher’s transform to conduct group-analyses.

#### Statistics

First, one-sample t-tests were calculated separately for each group to identify significant iFC between the ROIs. Next, two-sample t-tests were performed to identify between-group differences in iFC. Finally, brain-behavior multiple regression analyses were conducted to identify functional connections for which iFC positively correlated to emotion recognition performance. All models included the following nuisance regressors: head mircro-motion (mean FD), performance IQ, age and sample (adolescent sample or adult sample). All ROI-to-ROI iFC analyses were thresholded at p < 0.05, corrected for multiple comparisons (false discovery rate (FDR)).

## Results

### Behavioral performance

ANOVA analyses were conducted to explore group differences (ASD, TC) in behavioural performance on the emotion and control task. Group differences in **performance index** (accuracy/reaction times) were revealed on the emotion recognition task [Tukey, p = 0.01] [Cohen's d = 4.76]. No such group differences were revealed on the control task [p = 0.87] [Cohen's d = 1.15] (Fig 1B). This was revealed by a significant ‘group x task’ interaction effect [F(1, 56) = 6.95, p = 0.01].

A similar pattern of results was revealed for the **accuracy** [‘group x task’: F(1, 56) = 4.14, p = 0.05] and **reaction time** measures [‘group x task’: F(1, 56) = 5.49, p = 0.02] separately (S2 Fig).

### Graph theoretical network analysis using a fixed cost threshold

#### Group differences

**Fig 2:**
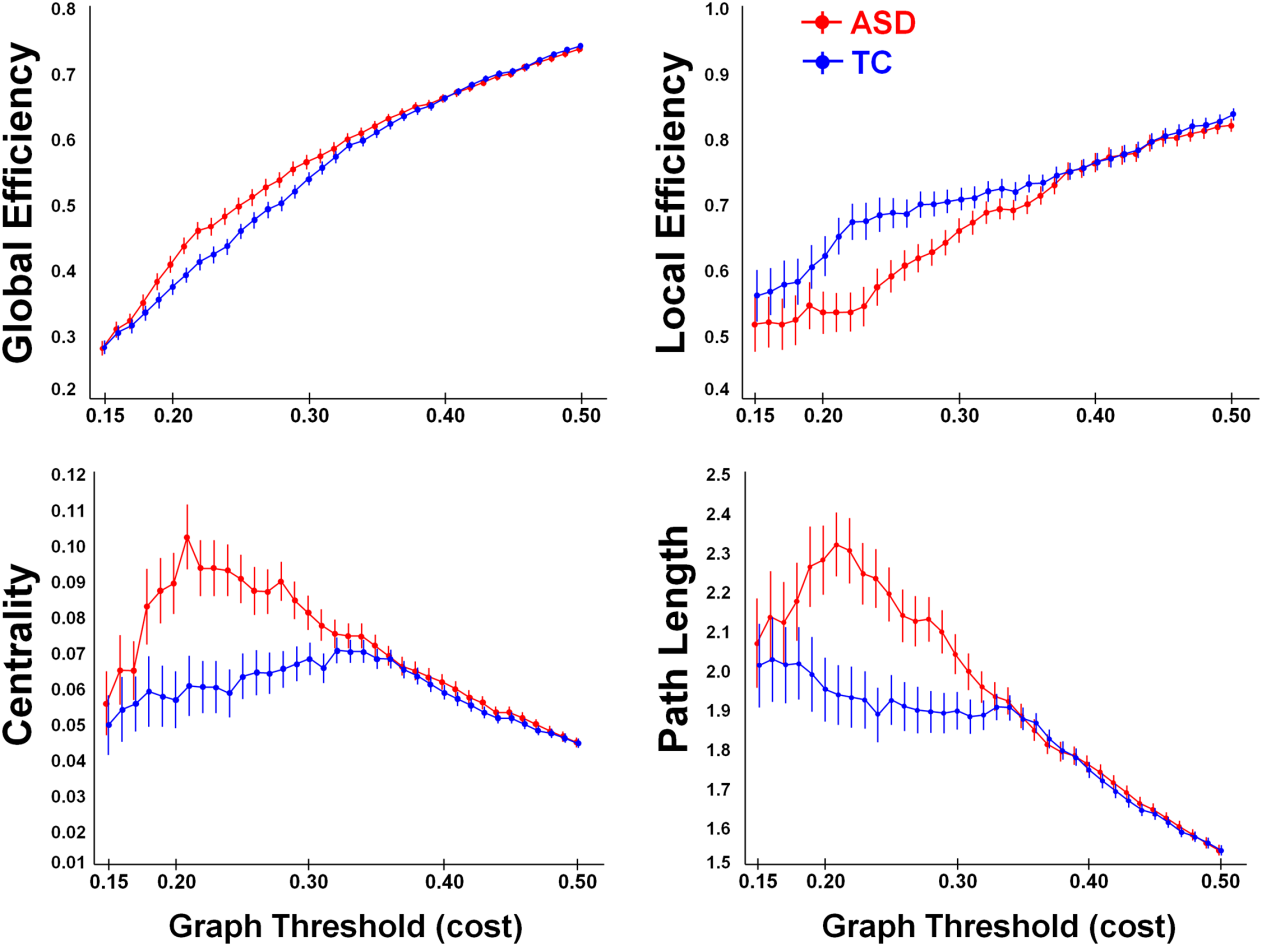
Graph theoretical network analysis using fixed cost thresholds (# 36, between *k* = 0.15 and *k* = 0.50). At the low-range cost thresholds (0.20 < *k* < 0.30), the TC-group (in blue) showed significantly higher local efficiency, shorter average path length and a lower betweenness-centrality, compared to the ASD group (in red). No group differences were revealed at the high cost thresholds (*k* > 35).

Across all examined cost thresholds (# 36, between *k* = 0.15 and *k* = 0.50), we revealed significant main effects of ‘group’ for the graph measures ‘local efficiency’ [F(1, 52) = 4.96; p = 0.03], ‘betweenness-centrality’ [F(1, 52) = 9.82; p < 0.01] and ‘path length’ [F(1, 52) = 9.51; p < 0.01]. A tentative group effect was revealed for ‘global ‘efficiency’ [F(1, 52) = 3.47; p = 0.06]. These main effects of ‘group’ were however significantly modulated by the applied cost threshold as revealed by significant ‘group x threshold’ interaction effects for all graph measures [global efficiency (F(35, 1820) = 3.70, p < 0.001); local efficiency (F(35, 1820) = 2.50, p < 0.001); betweenness-centrality (F(35, 1820) = 3.58, p < 0.001); path length (F(35, 1820) = 3.07, p < 0.001)]. As seen in Fig 2, the interaction effects indicated that group differences were predominantly evident for the low-range cost thresholds (between *k* = 0.20 and *k* = 0.30, approximately), whereas for the high cost thresholds (*k* > 35), differences between groups were largely absent. In other words, when only a limited number of connections were allowed to enter the graph network (at the low-range cost thresholds), the TC-group showed tighter functional network organization compared to the ASD-group, manifested in higher local efficiency, shorter average path length between the network nodes and a lower betweenness-centrality (Fig 2). However, when increasingly more connections are imposed to enter the graph network (at the high cost thresholds), network topology appeared to be overall similar for the ASD and TC groups.

#### Brain-behavior relationship

Brain-behavior correlations were performed to test whether graph measures were correlated with emotion recognition performance (performance index). Across all examined cost thresholds (# 36), we revealed no significant brain-behavior relationship for any of the graph measures [all, F(1, 52) < 0.11; p > 0.73]. For the graph measure ‘local efficiency’, a non-significant trend towards a ‘performance x threshold’ interaction was revealed [F(35, 1820) = 1.36; p = 0.07], indicating that at the lowest cost thresholds (*k* = 0.15; *k* = 0.16 and *k* = 0.17), tentative negative relationships with emotion recognition performance were evident (i.e., indicating that increased local efficiency was predictive of poor emotion recognition performance). Note however that these correlations were mild and did not survive corrections for multiple comparisons [all p > 0.05].

#### Identification of network hubs

**Fig 3:**
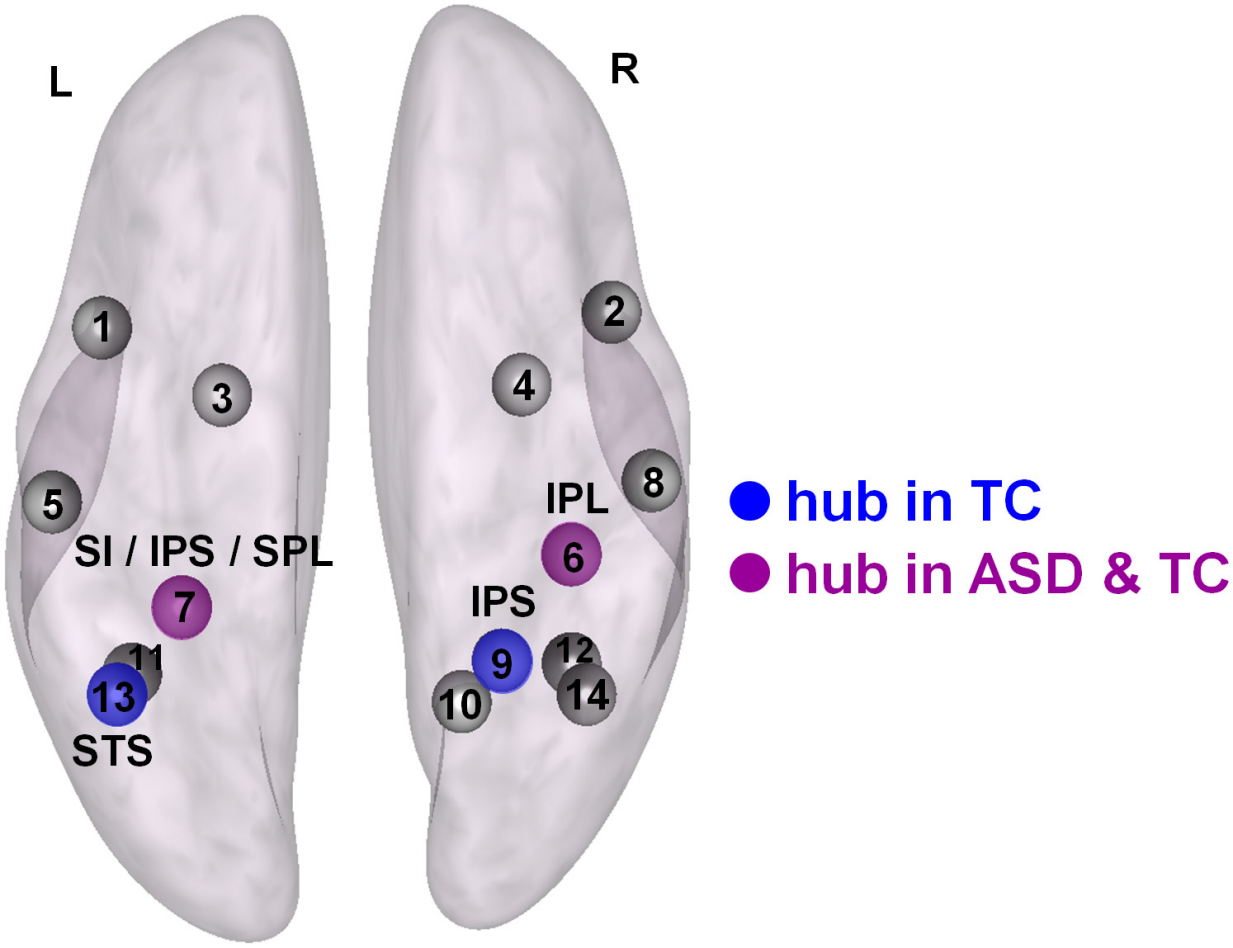
Identification of network hub nodes based on the graph theoretical network analysis using fixed cost thresholds. Hubs identified in both groups (ASD and TC) are indicated in purple. Hubs identified only in the TC group are indicated in blue. No hubs were identified only in the ASD group. L: left, R: right, IPL: inferior parietal lobule, SI: primary somatosensory cortex, IPS: intraparietal sulcus, SPL: superior parietal lobule, STS: superior temporal sulcus. Numbers correspond to the regions listed in Table 2.

Nodes with the largest betweenness-centrality were identified as ‘hubs’ in the network if the values of nodal betweenness were 2 SDs greater than the average betweenness-centrality of the entire network. In both the ASD and TC groups, the left intraparietal sulcus node (IPS, # 7 in Table 2) and the right inferior parietal lobule node (IPL, # 6) were identified as hub nodes (at *k* > 0.30) (indicated in purple in Fig 3). Additional hubs were identified in the right intraparietal sulcus node (IPS, # 9) and the left superior temporal sulcus node (STS, # 13) (at *k* > 0.35), but these were only evident in the TC group, not in the ASD group (indicated in blue in Fig 3).

### Graph theoretical network analysis using a fixed correlation threshold

#### Group differences

**Fig 4:**
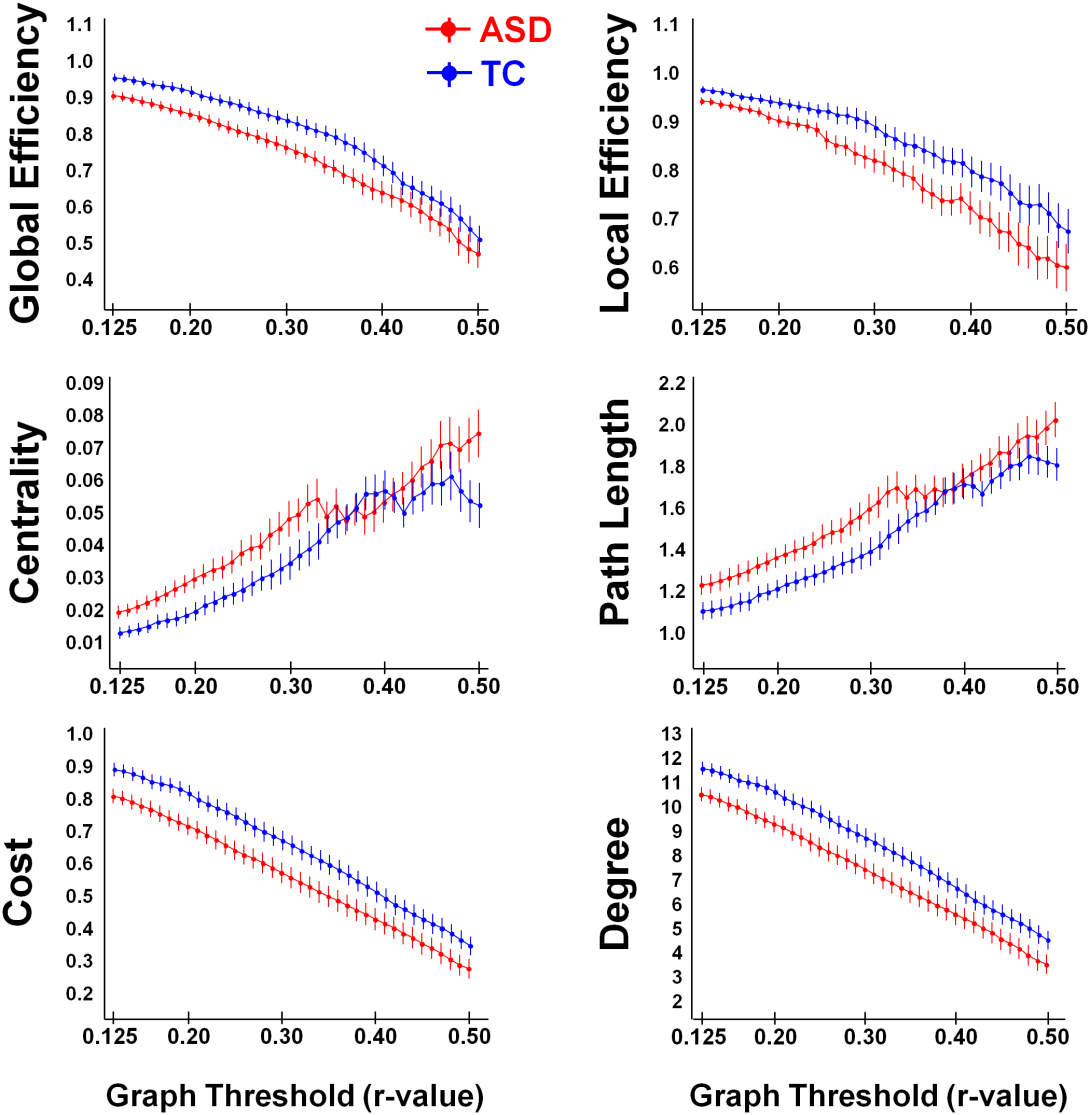
Graph theoretical network analysis using fixed correlation values (# 39, between *r* = 0.12 and *r* = 0.50). Across all thresholds, the TC-group (in blue) showed significantly higher global efficiency and local efficiency; reduced average path length and betweenness-centrality; and higher network density (cost) and mean connectivity degree, compared to the ASD group (in red).

Across all examined correlation thresholds (# 39, between *r* = 0.125 and *r* = 0.50), the TC-group consistently showed tighter functional network organization, manifested in a higher mean connectivity degree and network density (cost), higher global and local efficiency, shorter average path length between the network nodes and a lower betweenness-centrality (Fig 4). This was revealed by significant main effects of ‘group’ for all graph measures [all, [F(1, 52) > 3.80; p < 0.05]. Importantly, no significant ‘group x threshold’ interaction effects were revealed [all, F(38, 1976) < 1.00; p > 0.40] (Fig 4), indicating that effects of group consistently emerged irrespective of the examined correlation value threshold (# 39). Further, note that the direction of group differences in local efficiency, betweenness-centrality and path length was similar for the correlation value threshold (Fig 4) and cost threshold analysis (Fig 2, low-range cost values).

#### Brain-behavior relationship

With graph network density not equated across individuals, we revealed no significant relationships between emotion recognition performance (performance index) and any of the graph measures [all, F (1, 52) < 0.22; p > 0.6]. This was shown irrespective of the examined graph threshold [emotion task performance x threshold interaction: F(38, 1976) < 1.00; p > 0.4].

#### Identification of network hubs

No network hubs were identified in the ASD or TC groups for any of the examined correlation value thresholds.

### ROI-to-ROI intrinsic functional connectivity

**Fig 5:**
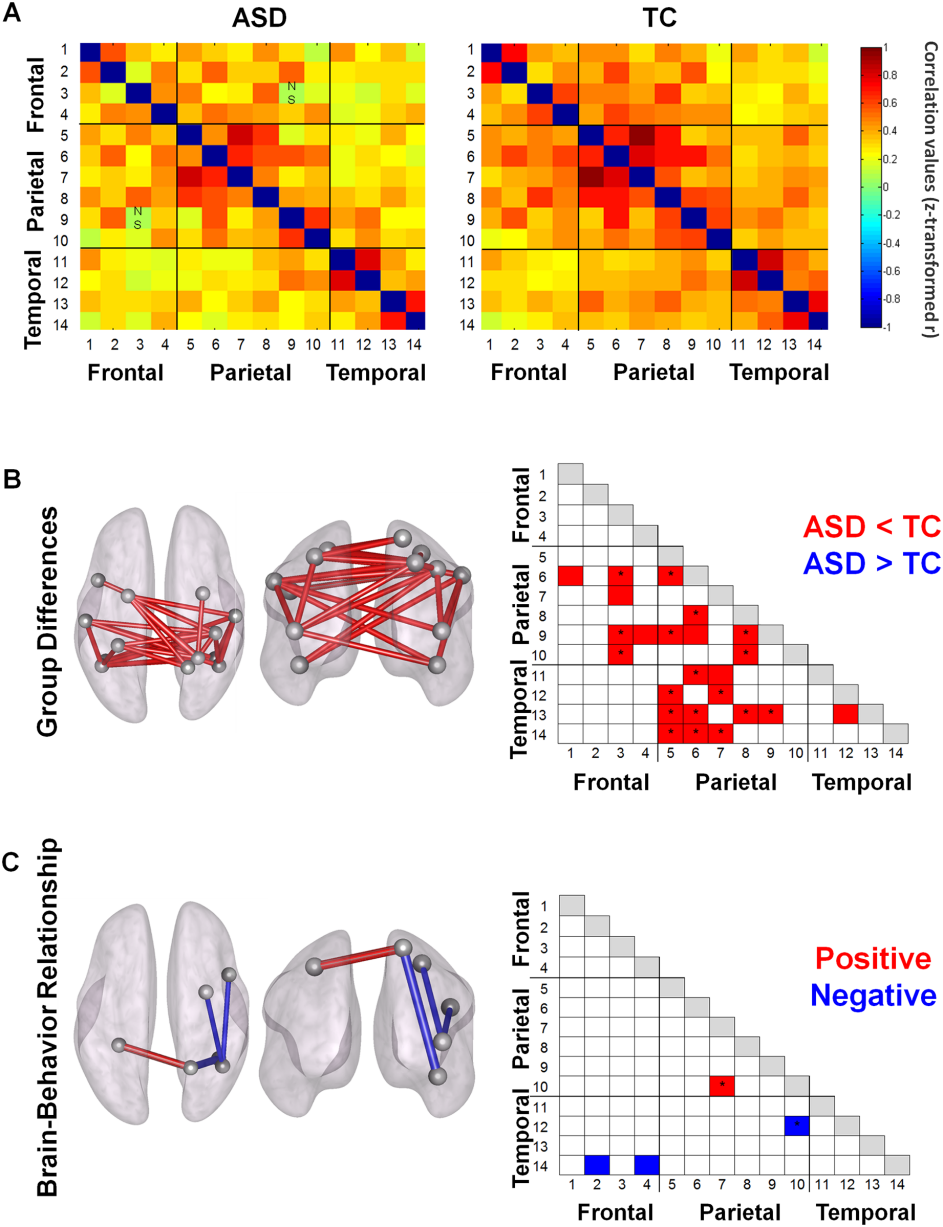
ROI-to-ROI intrinsic functional connectivity (iFC). (A) ROI-to-ROI correlation matrices for the ASD- and TC-groups. Normalized correlation coefficients are shown for each of the 14 x 14 ROI correlations for each group. Each row/column is labeled with a number which corresponds to one of the 14 ROIs reported in Table 2. (B) Between-group comparisons showed significant hypo-connectivity in the ASD-group (ASD < TC). None of the connections showed hyper-connectivity in the ASD-group (ASD > TC). (C) Only a few ROI-to-ROI connections showed a significant relationship with behavioral performance on the emotion recognition task. Particularly for the right superior parietal lobule (SPL, #10), a positive (with right SPL, #7) and negative (with right fusiform gyrus, #12) relationship was shown.

Both groups showed significant positive ROI-to-ROI iFC between nodes of the AON. Normalized correlation matrices are visualized in Fig 5A separately for each group (thresholded at p<0.05, FDR-corrected).

#### Group differences

Direct group comparisons revealed significant hypo-connectivity in ASD between several nodes of the AON. Only hypo- (ASD < TC) not hyper-connectivity (ASD > TC) was shown in ASD compared to TC even after the threshold for statistical significance was lowered to p < 0.05 uncorrected (for completeness, Fig 5B displays connections at an uncorrected p < 0.05 threshold, with a * indicating significant connections at a p < 0.05 FDR-corrected threshold). Overall, as shown in Fig 5B, hypo-connections were most pronounced between parietal and temporal nodes.

#### Brain-behavior relationship

Only a few significant brain-behavior relationships were revealed, particularly for right superior parietal lobule (SPL) (Fig 5C). A positive relationship indicated that high connectivity between right and left SPL was predictive of high performance on the emotion task, whereas a negative relationship for iFC between right SPL and right fusiform gyrus, indicated reduced performance with increasing connectivity (p < 0.05, FDR-corrected). At a p < 0.05 uncorrected threshold, negative brain-behavior relationships were also revealed for iFC between right STS and two frontal areas (right inferior frontal gyrus, right dorsal premotor cortex) (Fig 5C).

### Secondary Analyses

Primary findings were identified using a sample including both male (25 ASD, 28 TC) and female participants (2 ASD, 3 TC). Secondary analyses were performed excluding the female participants to verify consistency of the results. Overall, findings of the secondary analyses were qualitatively similar to the primary analyses. Particularly, the graph theoretical network analysis using **fixed cost thresholds** revealed similar main effects of ‘group’ [all F(1,47) > 6.20, p < 0.02] and significant ‘group x threshold’ interaction effects [all, F(35, 1645) > 3.30, p < 0.001], indicating that group differences in graph measures varied depending on the applied cost threshold. For the graph theoretical network analysis using **correlation value thresholds**, results of the secondary analyses were also highly similar to the primary analysis, indicating significant group differences in the graph measures [main effect of ‘group’, all, F(1,47) > 5.43, p < 0.03], irrespective of the examined correlation value threshold [non-significant ‘group x threshold’ interaction F(38, 1786) < 0.75, p > 0.6]. Also for the **ROI-to-ROI** functional connectivity analyses, primary findings of ASD-related hypo-connectivity (not hyper-connectivity) were replicated in the secondary analyses.

## Discussion

The present study investigated the functional network organization of the action observation network (AON) in ASD and TC by assessing graph theoretical measures and ROI-to-ROI intrinsic functional connectivity (iFC).

Compared to the ASD-group, TC showed higher functional network integrity as revealed by group differences in graph theoretical measures and ROI-to-ROI iFC. Particularly, findings of iFC hypo-connectivity, decreased graph network density (cost and degree) and efficiency in ASD, together with increased shortest path lengths and centrality consistently indicated that integrity of the AON is altered in ASD. While basic differences in empirical connectivity or density contributed to reductions in network integrity, graph analyses indicated that also the network-level organizational properties of the AON are altered in ASD.

Based on the graph theoretical analysis using a fixed correlation value threshold, our study revealed clear differences in network integrity of the AON between TC- and ASD-groups. At each of the adopted correlation value thresholds, the ASD-group displayed a reduced network density or fewer connections compared to the TC-group, indicating that overall lower connectivity strengths significantly reduced integrity of the AON in ASD. Especially connections between parietal and temporal nodes appeared to be altered, as revealed from the regional ROI-to-ROI iFC analysis. Overall, differences in network density were paralleled by an apparent loss of efficient information transfer within the AON in the ASD-group as revealed by larger average path lengths and reduced global efficiency. These findings are consistent with prior EEG studies, also demonstrating lager average path lengths in toddlers [70] and adults with ASD [71]. Additionally, findings of increased betweenness-centrality in ASD-participants are consistent with them having longer mean path lengths between network nodes compared to the TC-group. Particularly, shorter path lengths are a manifestation of a more direct connection between network nodes, resulting in less nodes participating in shortest paths between other network nodes. Considering that betweenness-centrality only quantifies the number of shortest paths that travel through each node, betweenness of the whole network will be reduced in participants with short path lengths (as seen in the TC-participants).

Similar to the graph analysis with a fixed correlation threshold, graph analysis with a fixed density or cost threshold (i.e., imposing an equal number of connections in each participant) showed reductions in local efficiency, larger average path lengths and increased centrality in the ASD group, at least for low-range cost thresholds. These findings provide indications that, in addition to the observed overall reductions in network density; also the basic network topology of the AON may be altered in ASD. Particularly, also when adjusting for overall differences in empirical connectivity strength, participants with ASD continued to display reductions in network integrity, suggesting that also the network-level organizational properties of the AON may be different in ASD and TC groups. The differential identification of hub nodes in the right inferior parietal lobule and left superior temporal sulcus only in the TC group, not in the ASD group are in line with this observation. These hub regions were also shown to display profound hypo-connectivity in the ASD compared to the TC group based on the ROI-to-ROI connectivity analysis, suggesting that these nodes may be key regions in altering the overall network topology of the AON in ASD.

Although complementary, specific advantages and disadvantages can be noted for graph theoretical approaches using either a fixed correlation or a fixed cost threshold [72]. The approach of a fixed correlation value threshold includes for each subject only those connections that exceed the imposed threshold, hence resulting in graphs with different numbers of connections or different densities across subjects. While these inter-individual differences in network density often reflect real and important differences in network topology across participants and particularly across groups, this approach may yield size-dependent effects on the calculated graph measures. The approach of a fixed cost or density threshold, on the other hand ensures an equal number of connections across participants. While choosing matrices with equal size and density avoids potential size-induced biases, this approach may also manipulate the empirical network topology by over- or underrating connections. Especially for groups where the overall empirical connectivity varies profoundly (as revealed for the AON in ASD in this study), one should be cautious with the use of fixed densities, as for participants with a *‘high’* average connectivity, connections that are important may be ignored because including them in the adjacency matrix would result in a density exceeding the matrix threshold, whereas, for participants with a *‘low’* average connectivity, a number of ‘low’ connections may be included in the adjacency matrix in order to achieve the imposed density threshold, despite the possibility that these connections are in fact ‘non-significant’ or irrelevant. In our cost thresholded graph analysis, we showed that group differences in graph measures were largely absent when increasingly higher cost thresholds were imposed. While this finding suggests similar network topologies in ASD and TC groups at high cost thresholds, the possibility cannot be ruled out that a number of low, ‘non-significant’ connections were imposed to enter the graph network, at least for the ASD group. Overall, when comparing patient groups with potential variations in overall network density it seems recommendable to evaluate graph matrices over a large range of thresholds, using both fixed correlation value and fixed cost or density thresholds.

Aside the exploration of group-related effects, a principal aim of this study was to examine brain-behavior relationships between measures of functional network organization and behavioral performance on a bodily emotion recognition task based on point light displays [22,32,33]. Conceptualizing autism-relevant indices as a continuum, rather than representing autism symptoms as a category is relevant in terms of the most recent diagnostic viewpoints incorporated in the DSM-V and accords with the recent NIMH Strategic Plan for Research Domain Criteria (R-Doc), classifying psychopathology based on dimensions of observable behavior and neurobiological measures. The use of PLDs in previous autism studies have proven highly advantageous for discriminating behavioral differences between ASD and TC, and revealing subtle inter-individual variations. Also in the present study, robust ASD-TC group differences on behavioral emotion recognition performance were revealed. However when correlating behavioral performance with the examined graph theoretical measures, no clear brain-behavior relationships were revealed. Only from the network analysis with low-range fixed cost or density thresholds, increased whole network ‘segregation’ (local efficiency) was predictive of decreased emotion recognition performance. Correlations were however mild and could not be reproduced at higher matrix thresholds. The absence of consistent brain-behavior relationships in the present study is at odds with results from a previous study from our lab, showing robust correlations between emotion recognition performance and the extent of neural recruitment of the STS (both in terms of task-based activations and functional connectivity with fronto-parietal regions) [22]. Considering these divergent findings, future work is needed to explore whether inter-individual variations in the visual perception of bodily motion/ emotions and related altered neural responses can provide useful scales for incorporating a dimensional perspective on autism.

To conclude, this work provides evidence of reduced functional connectivity in ASD, significantly reducing network density and integrity of the AON in ASD. In addition to the observation of overall reductions in empirical connectivity, graph theoretical analysis also provided indications that network-level organizational properties of the AON are altered in ASD.

## Supporting Information

S1 Fig Head motion. Mean frame-wise displacement (mean FD) was assessed for each participant over the entire resting-state fMRI scan. Each individual's value and group mean and standard deviations are displayed. Mean FD was not significantly different between groups (ASD, TC) (t(56) = -.07; p > .05) and did not exceed 0.5 mm in any of the participants.
(PDF)

S2 Fig Accuracy and reaction times on the emotion recognition and control test. Group differences in accuracy and reaction times were evident on the emotion recognition task, indicating higher accuracy and lower reaction times in TC, compared to ASD. No such effects were revealed for the control task.
(PDF)

S1 Movie One trial of the emotion recognition task. The emotional state of the blue-bordered point light display (PLD) had to be indicated relative to a baseline PLD (yellow-bordered) that always showed a neutral emotional state. The emotional state could be indicated as happy, sad, angry, or neutral (sad in this example).
(WMV)

S2 Movie One trial of the control task. Point light displays (PLD) of the **control task** were identical to those presented during the emotion recognition task, except participants were required to indicate color changes in the moving point lights. One of the dots in the yellow-bordered PLD briefly changes color to either red or green and participants had to indicate the number of dots (0-1-2-3) that changed into the same color in the blue-bordered PLD (2 in this example).
(WMV)

S1 Table Group characteristics for the ASD and TC participants (adolescents and adults separately). (PDF)
