# Supplementary material for: Functional Organization of the Action Observation Network in Autism: A Graph Theory Approach
Source: PLoS One. 2015 Aug 28;10(8):e0137020. doi: 10.1371/journal.pone.0137020 (PMC4552824; doi:10.1371/journal.pone.0137020)

S1 Figure

Head motion.

Mean frame-wise displacement (mean FD) was assessed for each participant over the entire resting-state fMRI scan. Each individual's value and group mean and standard deviations are displayed.

Mean FD was not significantly different between groups (ASD, TC) ($t(56) = -.07$; $p > .05$) and did not exceed 0.5 mm in any of the participants.

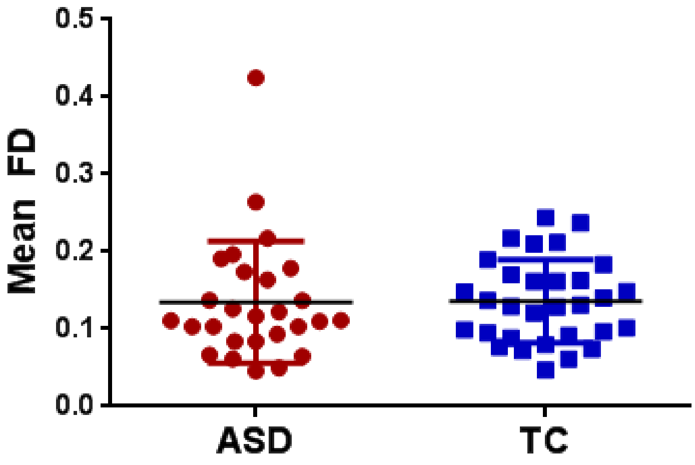

Supplement: S1 Fig — Mean frame-wise displacement (mean FD) was assessed for each participant over the entire resting-state fMRI scan. Each individual's value and group mean and standard deviations are displayed. Mean FD was not significantly different between groups (ASD, TC) (t(56) = -.07; p > .05) and did not exceed 0.5 mm in any of the participants. (PDF) [file pone.0137020.s001.pdf]
