# Supplementary material for: Functional Organization of the Action Observation Network in Autism: A Graph Theory Approach
Source: PLoS One. 2015 Aug 28;10(8):e0137020. doi: 10.1371/journal.pone.0137020 (PMC4552824; doi:10.1371/journal.pone.0137020)

S2 Figure

Accuracy and reaction times on the emotion recognition and control test.

Group differences in accuracy and reaction times were evident on the emotion recognition task, indicating higher accuracy and lower reaction times in TC, compared to ASD. No such effects were revealed for the control task.

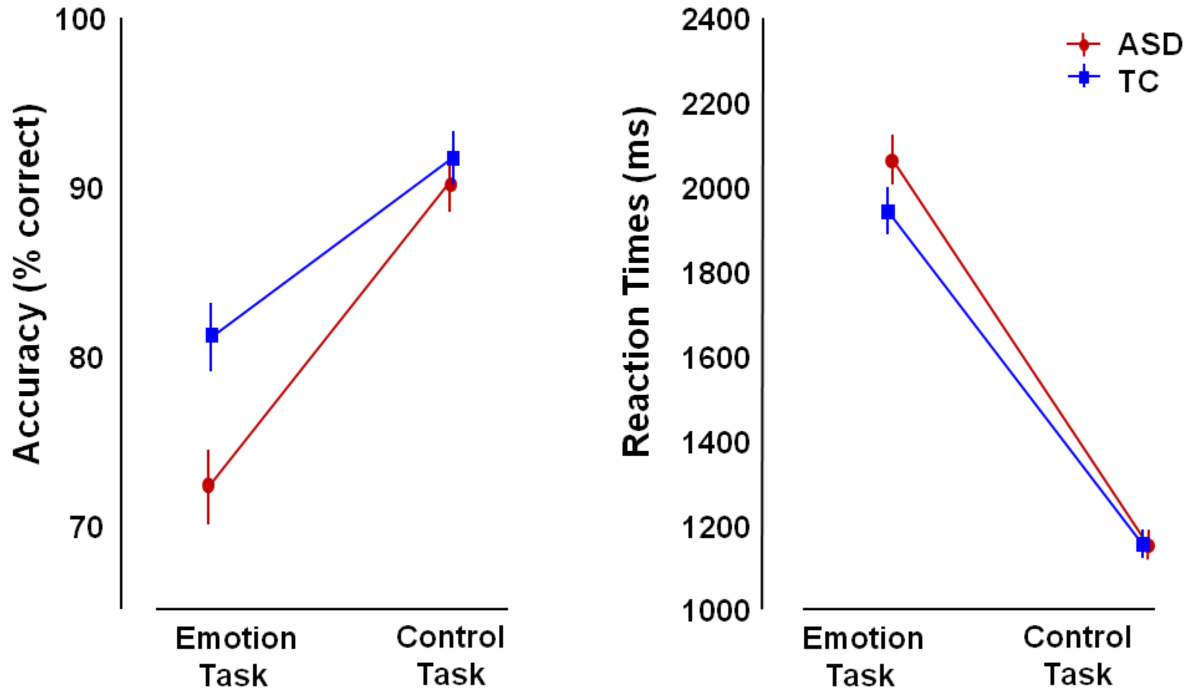

Supplement: S2 Fig — Group differences in accuracy and reaction times were evident on the emotion recognition task, indicating higher accuracy and lower reaction times in TC, compared to ASD. No such effects were revealed for the control task. (PDF) [file pone.0137020.s002.pdf]
