# Supplementary material for: Functional Organization of the Action Observation Network in Autism: A Graph Theory Approach
Source: PLoS One. 2015 Aug 28;10(8):e0137020. doi: 10.1371/journal.pone.0137020 (PMC4552824; doi:10.1371/journal.pone.0137020)
Supplement: S1 Table — (PDF) [file pone.0137020.s005.pdf]

S1 Table

Group characteristics for the ASD and TC participants (adolescents and adults separately).

	Adolescents				Adults			
	ASD (n=12)		TC (n=16)		ASD (n=15)		TC (n=15)	
Sex	10 males		13 males		All males		All males	
	**mean**	**SD**	**mean**	**SD**	**mean**	**SD**	**mean**	**SD**
SRS Scores	95.1	28.0			91.5	28.5		
Age (years)	13.8	1.1	14.2	1.6	21.7	4.0	23.3	2.9
Verbal IQ	90.3	15.5	115.8	12.3	109.1	12.9	117.4	9.9
Performance IQ	100.3	11.2	106.7	8.8	105.6	19.3	109.1	17.7
Head Motion (mean FD)	0.18	0.09	0.17	0.04	0.09	0.04	0.10	0.04

ASD. autism spectrum disorder; TC. typical controls; SRS. Social Responsiveness Scale (raw Total); IQ. intelligence quotient; mean FD. mean framewise displacement; SD. standard deviation.
